# Scoping Review of Virtual Reality (VR)-Based Disaster Mitigation Education

**DOI:** 10.7759/cureus.74062

**Published:** 2024-11-20

**Authors:** Ryohei Kimura, Ayako Fukushima, Kohei Kajiwara, Hideaki Sakuramoto, Shun Yoshihara, Kimie Harada, Teruyuki Nakayama, Akiko Ito

**Affiliations:** 1 Faculty of Nursing, Japanese Red Cross Kyushu International College of Nursing, Munakata, JPN; 2 Faculty of Nursing, Japanese Red Cross Toyota College of Nursing, Toyota, JPN

**Keywords:** disaster, education, learning, mass casualty incidents, virtual reality

## Abstract

This study explored the use of virtual reality (VR) in disaster preparedness education, focusing on VR scenarios, disaster types, and user interactivity to identify gaps in existing research. A scoping review methodology, based on the Arksey and O'Malley framework and Preferred Reporting Items for Systematic Reviews and Meta-Analyses-Scoping Reviews (PRISMA-ScR) guidelines, was used, and the protocols were registered in the UMIN Clinical Trials Registry (UMIN000052800). The review included PubMed, CINAHL, the Cochrane Central Register of Controlled Trials in the Cochrane Library, and Ichushi-Web of the Japan Medical Abstract Society, with data up to January 31, 2024. Studies on disaster preparedness and mitigation education using VR were included, without restrictions on study design, country, or language. We excluded studies utilizing non-immersive VR, as well as non-academic letters to the editor, editorials, commentaries, review articles, conference abstracts, and non-academic manuscripts. In the first screening phase, 2 researchers independently reviewed the titles and abstracts of 516 articles and conducted the screening. A total of 17 articles were selected. In cases where there were differing opinions on inclusion or exclusion, the two researchers discussed the matter together. In the second screening phase, the 17 selected articles underwent full-text screening. Three articles were excluded because their outcomes did not align with our research. Eight articles were excluded based on our predefined criteria, which included commentaries, editorials, and review articles. Ultimately, six articles were included in the meta-analysis. Of these, three studies were from East Asia, one from Australia, one from North America, and one from an unspecified region. Disaster types included chemical, radiation, explosion, fire, and earthquake events. The VR training scenarios covered evacuation, first aid, patient transport, triage, decontamination, and other skills. Five of the studies emphasized user interactivity and were primarily aimed at professionals such as medical staff and nursing students. Current VR disaster response training primarily targets professionals, demonstrating its effectiveness and importance; however, there is a lack of studies focusing on training for the general public, especially in the least developed countries. Extending VR-based training programs to the general public is critical to improving countries' disaster response capabilities.

## Introduction and background

Many natural disasters occur worldwide each year, causing tens of thousands of deaths. While the annual death toll due to disasters represents only a small fraction of the global total, the cumulative death toll is significant [1]. Vulnerable populations, especially those in low- and middle-income countries, constitute the majority of people affected by disasters [2,3]. More than 400 earthquakes with magnitudes exceeding five on the Richter scale have been recorded in the Asian region, as of April 2024. The most significant was the Noto Peninsula earthquake on January 1, 2024, in Ishikawa Prefecture, Japan, which measured 7.5 on the Richter scale, followed by the Taiwan earthquake on April 2, 2024, at 7.4 on the Richter scale [4,5].

Japan has implemented various disaster prevention and mitigation initiatives to address its vulnerability to earthquakes. However, following the Great Hanshin-Awaji Earthquake of 1997, the country’s approach shifted from disaster prevention, which aims to prevent disasters, to disaster mitigation, which emphasizes minimizing the damage caused by a disaster [6-8]. By considering local living standards, infrastructure, and response systems, more lives can be saved in geographically disaster-prone areas and low- and middle-income countries [9].

Given the unpredictable nature of disasters, preparedness is a crucial part of daily life, and disaster mitigation education plays a pivotal role in ensuring this. A previous study demonstrated that training improved disaster prevention and mitigation more than educational video screenings and controls, specifically among students in disaster-prone areas [10]. Another study highlighted that VR use in disaster preparedness training complements existing traditional approaches [11]. The immersive and participatory nature of VR training offers real-life, high-risk scenarios that are non-existent in traditional approaches. By utilizing VR, it becomes possible to re-create disaster scenarios that are difficult to replicate in reality. This allows users to undergo safe and more practical disaster training, which is highly significant.

Consequently, VR technology is becoming increasingly valuable in disaster preparedness training, owing to its cost advantages over large-scale live training [11]. The key differences from traditional training methods are that, by utilizing VR, users can train in a safe environment, repeat experiences as needed, and engage in training at any time that suits them. Additionally, VR allows for the re-creation of various disaster scenarios, making it possible to tailor the experience to meet the specific needs of the users, offering numerous benefits. Farra et al. emphasized that disaster training is crucial for reducing both mortality and morbidity and that the use of VR simulation for such training has the potential to be a powerful educational tool [12]. Currently, several VR-based training programs are being developed to either replace or complement current traditional approaches to disaster preparedness training. Several extant studies have demonstrated the usefulness of this technology [13-15]. This change in disaster mitigation education also indicates that more effective education methods are achievable. Understanding global trends and identifying current issues in disaster prevention and mitigation education using VR can lead to the development of such effective methods. Technology-based educational support is useful in terms of location and cost. Therefore, because disaster damage in low- and middle-income countries is typically severe, finding new methods for disaster prevention and mitigation to minimize damage will create effective response systems.

In this study, we hypothesize that the role of professionals in global disaster mitigation is significant. Therefore, with the current global increase in large-scale natural disasters, it is vital to understand global trends and develop effective VR-based disaster mitigation education. Focusing on identifying research gaps, this study reviews current literature on VR use in disaster mitigation education, VR scenarios and types of disasters, and user interactivity in VR programs, using the scoping review method. Specifically, the research question we aim to answer is, “How is VR being utilized for disaster mitigation education globally?”

## Review

Methods

We conducted a scoping review to comprehensively explore and map the reality of disaster mitigation education that uses VR. We applied the standard framework reported by Arksey and O’Malley [16,17] and further extended by the Joanna Briggs Institute [18]. We also adapted the reporting guidelines described in the Preferred Reporting Items for Systematic Reviews and Meta-analyses Statement (PRISMA), an extension of the PRISMA Scoping Reviews (ScR) [19]. The protocol was registered in the UMIN Clinical Trials Registry (UMIN-CTR: UMIN000052800). Disaster mitigation education was defined as education aimed at preventing disasters from occurring and reducing the damage when a disaster occurs. Ethical approval was not required for this review, as it involved a secondary analysis of existing published data.

Identifying Relevant Studies

A comprehensive search was conducted across multiple databases, including PubMed, CINAHL, the Cochrane Central Register of Controlled Trials in the Cochrane Library, and Ichushi-Web of the Japan Medical Abstract Society. The search terms used are given in the Appendices. The search spanned the inception of each database to January 31, 2024. Additionally, relevant studies from the reference lists of identified articles and manual searches of key journals were reviewed. The search formula was initially created in PubMed and subsequently adapted for use in the other databases. RK and AF conducted the initial search in collaboration with a librarian. The inclusion criteria were defined according to the eligibility determined by the disaster mitigation education specialist (details are described in the database.) We employed the Participants, Concept, and Context (PCC) framework [20], representing population-general citizens, disaster-related professions, and educators; concept education using VR; and context-disaster mitigation. The inclusion criteria were as follows: (1) research on disaster mitigation and mitigation education using VR; (2) no limitation on the type of disaster; (3) no limitation on research design; (4) no limitation on country; and (5) no limitation on language. We excluded studies utilizing non-immersive visual materials, such as those where participants only view videos or images on a monitor, as well as non-academic letters to the editor, editorials, commentaries, review articles, conference abstracts, and non-academic manuscripts.

**Table 1:** Inclusion and exclusion criteria for this scoping review VR: Virtual reality

Criteria	Details
Inclusion criteria	Studies focused on disaster mitigation and mitigation education using VR
	No restriction on the type of disaster
	No restriction on study design
	No restriction on the country of origin
	No restriction on language
Exclusion criteria	Studies involving non-immersive VR
	Non-academic sources such as letters to the editor, editorials, commentaries, review articles, conference abstracts, and non-academic manuscripts

Study Selection Process

**Figure 1:**
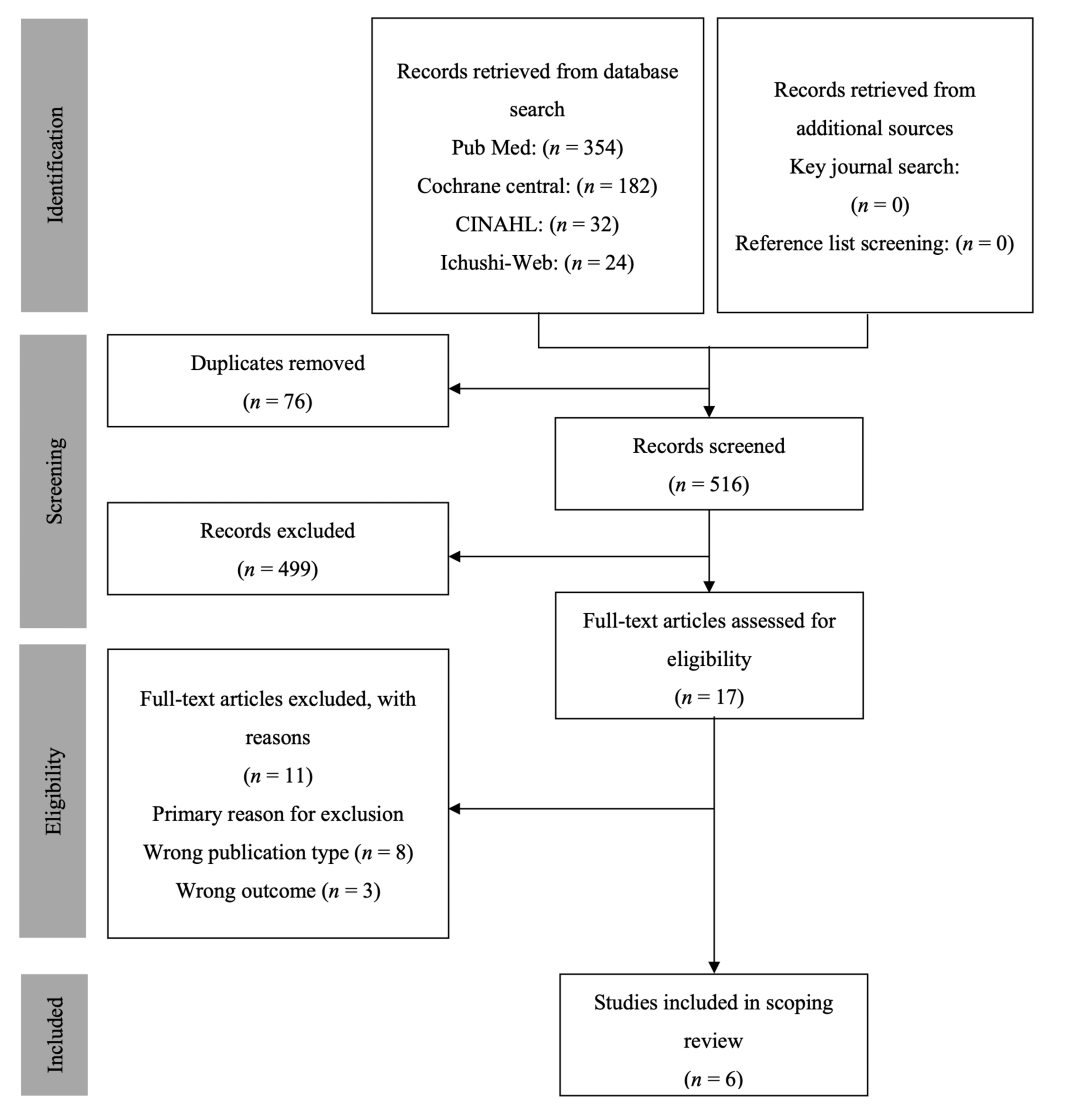
PRISMA flowchart of the study selection process PRISMA: Preferred Reporting Items for Systematic Reviews and Meta-Analyses

For the first screening, two reviewers (RK and AF) independently assessed the titles and abstracts of the extracted articles, after which a second screening involved a full-text screening against the eligibility criteria. Discrepancies in study selection between the two reviewers were resolved by discussion between them, as well as the other authors (KK, HS, SY, KH, and TN). Figure 1 illustrates the study selection process.

Charting the Data

The primary outcome of the scoping review for this study was “How is VR being used in disaster education worldwide?” Data from each study were extracted independently by one reviewer and reviewed by a second reviewer. The following information was extracted for each included article: first author, year of publication, country of publication, research problem and objectives, research design, subject matter, participants, VR content, outcome measures, and disaster mitigation education outcomes. The data were extracted from the original articles using a specially designed form and entered into a spreadsheet.

Results

Overview

A total of 592 records were retrieved based on the database searches. The additional searches did not yield any new records. After removing duplicates, 516 abstracts were screened for eligibility, after which 17 full-text articles were assessed. Three articles were excluded because their results did not align with the research outcomes we aimed for. Eight articles were excluded based on predefined criteria such as commentaries, editorials, and review articles. Six articles were ultimately included for qualitative synthesis.

Study Characteristics

Table 2 shows the characteristics, study designs, and outcome measures of the six studies. Five of these studies were randomized controlled trials [21-25]. One case was a before-after study [26]. Three studies were conducted in East Asia [21,25,26], one in Australasia [24], and one in North America [22]; the geographical coverage of the remaining study is unknown [23]. The participants were nursing students in three cases [22,25,26], healthcare workers in two cases [21,22], and junior secondary school students in one case [24].

**Table 2:** Characteristics of the included studies (N = 6) VR: Virtual reality, ER: Emergency room, RCT: Randomized control trials, VRS: Virtual reality simulation, CU: Clinical updates

Author (Year), Country, [Ref. No.]	Research aim	Study design	Subject	Procedures		Number of participants		Outcome measure
				Intervention group	Control group	Intervention group	Control group	
Chang et al. (2022), Taiwan, [20]	The effectiveness of a “360º virtual reality (VR) chemical disaster training program” on disaster preparedness and self-efficacy in emergency room (ER) nurses	RCT	ER nurses from two different hospitals	Chemical disaster training through 360º VR	Training through tabletop drills	32	35	SCDPI (Self-assessment of Chemical Disaster Preparedness Inventory), GSEF (General Self-Efficacy Scale)
									Farra et al. (2013), USA, [21]	The effects of VR simulation (VRS) on learning outcomes and retention of disaster training	RCT	Nursing students in their final year of study in either the capstone courses or pediatrics courses	Web-based disaster training and a disaster VRS	Web-based disaster training	22	25	Knowledge assessment through simulated disaster experiences
																		Farra et al. (2019), not specified [22]	The differences in learning outcomes and retention among healthcare workers who underwent VRS evacuation training for neonates and those who reviewed clinical updates (CU) with the same content as the VRS	RCT	Newborn intensive care unit (NICU) workers who are registered nurses, monitor technicians (unlicensed personnel), respiratory therapists, physicians, and advanced practice registered nurses	Web training in neonate evacuation and VRS	Web training in neonate evacuation and CU	Total 93 (randomly divided into VRS or CU treatment groups with stratified randomization by pre-assessment score results and job classification)		EPIQ (Emergency Preparedness Information Questionnaire), Evacuation Skills, Cognitive assessment, VARK (V: visual, A: aural, R: read/write, and K: kinesthetic)
Feng et al. (2021), Australasia [23]	The effects of immersive VR (IVR) and serious games (SGs) for earthquake training targeting children	RCT	Junior secondary school students (ages 11 to 15)	IVR SG training (a: immediate feedback, b: prior instruction, c: post-game assessment)	A leaflet	a: 31, b: 32, c: 31	31	Safety knowledge, self-efficacy																		
									Shujuan et al. (2022), China, [24]	The impact of VR scenarios on disaster preparedness among nursing students	RCT	Nursing students who had registered for the disaster nursing course	The usual disaster training and VR training scenarios	The usual disaster training	49	52	Disaster preparedness									
																		Hu et al. (2022), China, [25]	The effectiveness of a VR mobile game-based application for teaching disaster evacuation management education to nursing students	Before–after study	Nursing students who chose the Help and Rescue curriculum	Lecture and demonstration, virtual reality mobile game-based application	Lecture and demonstration	85	82	Educational knowledge and decision-making abilities

Scenario Content and Interactivity

Table 3 shows the types of disasters, scenario contents, and interactions under the VR scenarios. Three disaster types were fires [23,25,26], three were earthquakes [24-26], and two were either chemical, biological, or explosive disasters [21,22]. Four scenarios were dedicated to safe evacuation from the disaster scene [23-26]; three focused on medical procedures, including first aid during the evacuation, patient transport training, triage, debridement, and learning tracheal intubation techniques [21,22,25]. Five of the six studies included interactions or similar descriptions [21,22,24-26].

**Table 3:** Virtual reality (VR) scenarios *Interaction: Yes; Interactions and those with similar descriptions

Author (year)	Type of disaster	Scenario contents of VR	Interaction^＊^
Chang et al. (2022) [20]	Chemical disaster	Play the role of incidence commander, primary triage personnel, decontamination zone personnel, and secondary triage personnel	Yes
Farra et al. (2013) [21]	Radioactive and explosive events	Decontamination exercise, Start triage exercise	Yes
Farra et al. (2019) [22]	Fire	Horizontal evacuation of stable infants, Evacuation of positive pressure ventilation infants	Unknown
Feng et al. (2021) [23]	Earthquake	Goals for actions before, during, and after the earthquake	Yes
Shujuan et al. (2022) [24]	Fire Earthquake	Evacuation from earthquake and fire, Engage in firefighting, triage, wound dressing, fixation, hemostasis, debridement, cardiopulmonary resuscitation, tracheal intubation, transportation, decontamination, supportive psychological care	Yes
Hu et al. (2022) [25]	Fire Earthquake	Safety escape, First aid scenario during the evacuation	Yes

Discussion

This scoping review integrates the existing evidence on disaster mitigation education methods using VR, including evacuation from fire, initial response during earthquakes, chemical fires, and medical response to radioactive contamination. The review identified five relevant studies conducted across East Asia [21,25,26], Australasia [24], and North America [22] and one with an unknown geographical coverage [23]. Overall, disaster types included chemical, radiological, explosive, fire, and earthquake, and the VR scenarios were related to first aid during evacuation, patient transport training, and skills training such as triage, debridement, and tracheal intubation. Of these, five studies that used VR interactions were identified [21,22,24-26]. Most VR scenarios target professionals such as medical personnel [21,23] and nursing students [22,25,26].

The Importance of Expanding VR-Based Disaster Mitigation Education to the General Public

Disaster mitigation training using VR was initially intended for professionals, not the general public. Consequently, several highly effective programs have been created to educate professionals and students on how to respond to contingencies [27]. However, anthropogenic and natural disasters occur frequently and most disaster victims are non-professionals. Therefore, disaster prevention education for the public is essential and can be more effective if it is game-based [28]. Additionally, the private sector should become actively involved, rather than solely relying on governments and the military to minimize damage when disasters occur. Effective disaster prevention education for civilian responders can enhance disaster preparedness [29], and individual risk perception is critical to disaster management. Therefore, disaster prevention education should be available to both professionals and the general public. Disaster mitigation education is a functional, operational, and cost-effective tool for risk management. However, traditional live training is expensive, time-consuming, and unsafe [30-33]. Compared with untrained individuals, trained persons can effectively protect themselves. A previous study stated that planning and designing a comprehensive education program is necessary to help people face disasters [34]. Therefore, the widespread use of VR in disaster mitigation measures may safeguard the public from disasters and mitigate associated damages.

Global Disparities and the Need for Enhanced VR-Based Disaster Mitigation Training

Previous research has demonstrated the effectiveness of VR training for disaster prevention, particularly for professionals such as medical personnel [35]. Extant literature on disaster risk management is unevenly distributed across Southeast Asia, Australasia, and North America, whereas regions such as the Middle East, Africa, and South America lack sufficient studies. The Middle East faces risks from desertification and geopolitical factors, whereas Africa is vulnerable to floods and droughts. According to global data, South America is prone to earthquakes and volcanic activity [1]. Limited access to research and information may be the primary cause of the research gap. This is emphasized in a similar review revealing the lack of publications on disaster management in developing countries [9]. Other factors contributing to limited data in developing countries may include publication bias or a lack of research in the context of developing countries; this suggests the need to improve research quality and prioritize funding in these areas [36]. It is therefore important to promote research on disaster reduction in these regions and develop measures that address region-specific issues. Therefore, a comprehensive approach that considers the disaster reduction needs of each region from a global perspective is necessary. The scope and number of disasters have increased significantly over the years, prompting the need for more robust disaster response training approaches [37]. VR is capable of simulating real-life high-risk scenarios owing to its high immersive properties, making it an effective technique for disaster response training. Most studies on VR applications in disaster response training do not provide specific details regarding content scenarios. This could be owing to the distinct focus on the educational effects of VR scenarios. Further research is required to determine the most effective and diverse scenarios for disaster mitigation education.

## Conclusions

Based on the discussion, it is evident that a research gap persists in VR-based disaster mitigation education aimed at the general public. In the future, disaster mitigation education should be conducted across various regions to offer better insights into the effectiveness of VR-based disaster preparedness.

## Appendices

Search terms

PubMed

Disasters[mh] OR disaster*[tiab] OR hazard[tiab] OR emergenc*[tiab] OR mass casualty incident*[tiab] OR Mass Casualty Incidents[mh] OR mass casualty[tiab]

AND

virtual reality[mh] OR virtual realit*[tiab] OR vr[tiab] OR educational virtual realit*[tiab] OR instructional virtual realit*[tiab] OR Video Games[mh] OR video game*[tiab] OR computer game*[tiab]

AND

Education[mh] OR Teaching[mh] OR Learning[mh] OR education[tiab] OR workshop[tiab] OR teaching[tiab] OR teaching method[tiab] OR learning[tiab] OR seminar*[tiab] OR mass casualty training[tiab] OR simulation*[tiab] OR educational activit*[tiab] OR training program[tiab] OR training technique[tiab] OR training activit*[tiab] OR educational technique[tiab] OR Simulation Training[mh] OR simulation training[tiab] OR interactive learning[tiab]

NOT

animals [mh] NOT humans [mh]

The Cochrane Central Register of Controlled Trials in the Cochrane Library

Disasters[mh] OR disaster*[tiab] OR hazard[tiab] OR emergenc*[tiab] OR mass casualty incident*[tiab] OR Mass Casualty Incidents[mh] OR mass casualty[tiab]

AND

virtual reality[mh] OR virtual realit*[tiab] OR vr[tiab] OR Educational Virtual Realit*[tiab] OR Instructional Virtual Realit*[tiab] OR Video Games[mh] OR video game*[tiab] OR Computer Game*[tiab]

AND

Education[mh] OR Teaching[mh] OR Learning[mh] OR education[tiab] workshop[tiab] OR teaching[tiab] OR teaching method[tiab] OR learning[tiab] OR seminar*[tiab] OR mass casuality training[tiab] OR simulation*[tiab] OR educational activit*[tiab] OR rraining program[tiab] OR training technique[tiab] OR training activit*[tiab] OR educational technique[tiab] OR Simulation Training[mh] OR simulation training[tiab] OR interactive learning[tiab]

NOT

animals [mh] NOT humans [mh]

CINAHL

MH "Disasters" OR disaster* OR mass casualty incident* OR hazard OR emergenc* OR MH "Mass Casualty Incidents" OR mass casualty

AND

MH "Virtual Reality" OR MH "Video Games" OR virtual realit* OR VR OR video game* OR educational virtual realit* OR instructional virtual realit* OR computer game*

AND

MH "Education" OR MH "Seminars and Workshops" OR MH "Teaching" OR MH "Simulations" OR MH "Learning" OR MH "Mass Casualty Training" OR education* OR seminar* OR workshop OR teaching OR simulation* OR learning OR mass casualty training OR educational activit* OR training program OR training technique OR training activit* OR educational technique OR simulation training OR interactive learning OR teaching method

NOT

MH "Animals" NOT MH "Humans"

Ichushi - Web of the Japan Medical Abstract Society

災害/TH OR 被災/TA OR 減災/TA OR 防災/TA OR 災害/TA OR 多数傷病者事故/TH OR 多数傷病者事故/TA OR 集団外傷事故/TA OR 多重事故/TA OR 多数傷病者発生事故/TA OR 大量傷者事故/TA OR 緊急/TA OR 非常時/TA OR 非常事態/TA

AND

バーチャルリアリティー/TH OR バーチャルリアリティ/TA OR バーチャル・リアリティ/TA OR 仮想現実/TA OR 人工現実/TA OR ビデオゲーム/TH OR ビデオゲーム/TA OR TVゲーム/TA OR コンピューターゲーム/TA OR コンピュータゲーム/TA OR テレビゲーム/TA

AND

教育/TH OR 学習/TH OR 演習/TA OR シミュレーション/TA OR 教育/TA OR 訓練/TA OR 授業/TA OR 教授法/TA OR 教授活動/TA

NOT

動物/TH NOT ヒト/TH
